# Supplementary material for: Perceived warmth and competence predict callback rates in meta-analyzed North American labor market experiments
Source: PLoS One. 2024 Jul 10;19(7):e0304723. doi: 10.1371/journal.pone.0304723 (PMC11236140; doi:10.1371/journal.pone.0304723)
Supplement: S1 File — (PDF) [file pone.0304723.s001.pdf]

Supporting information

S1 Statistical definitions **The random effects model** The random-effects model accounts for between-study heterogeneity, which causes true effect sizes of studies to differ. Specifically, the model assume that $\hat{\theta}_k = \theta_k + \epsilon_k$, where $\hat{\theta}_k$ is the observed effect size, θ_k is the true effect size of study k, and ϵ_k is the sampling error. Furthermore, it assumes that the true effect size θ_k of study k is only part of an over-arching distribution of true effect sizes with mean μ: $\theta_k = \mu + \zeta_k$. Overall the random-effects model can be expressed as $\theta_k = \mu + \zeta_k + \epsilon_k$, indicating that the observed effect size deviates from the pooled effect μ because of two error terms, ζ_k and ϵ_k.

The pooled effect size is a weighted average of all studies. The weight w_k for each study k is calculated as the *inverse-variance*: $w_k^* = \frac{1}{s_k^2 + \hat{\tau}^2}$, where s_k^2 is the estimated within-study variance of the observed effect size, capturing the variability in study outcomes due to sampling error, and $\hat{\tau}^2$ is the estimated between-study variance, which accounts for the true effect sizes' heterogeneity across different studies.

To correct for our small samples, we adjust the model via the Knapp–Hartung modification [18,56,57], which, unlike the more common restricted maximum likelihood (REML) estimation, does not assume that the error distribution is normal. This technique adjusts the standard errors of the regression coefficients (including the intercept-only model, which calculates the meta-analytic effect size) by multiplying their variances by $q_{\mathrm{KH}} = \frac{\widehat{\boldsymbol{\theta}}\mathbf{P}\widehat{\boldsymbol{\theta}}}{K-p}$, with $\mathbf{P} = \mathbf{W}^* - \mathbf{W}^*\mathbf{X}\left(\mathbf{X}'\mathbf{W}^*\mathbf{X}\right)^{-1}\mathbf{X}'\mathbf{W}^*$, where $\mathbf{W}^* = \mathrm{diag}\left(w_1^*, w_2^*, \ldots, w_K^*\right)$, and $\mathbf{X} = \left(\mathbf{x}_1', \mathbf{x}_2', \ldots, \mathbf{x}_K'\right)'$ is the matrix of a vector from each study $k \in K$ of p moderators (including the intercept). Intuitively, this method incorporates the uncertainty of estimating τ^2, a factor that increases with smaller number of studies.

Correlation as an effect size For our analysis with names, we deploy the random-effects model to estimate the true correlation between PC1 and callback, ρ, with $r_k(\mathrm{callback}, \mathrm{PC1})$. The value r_k is transformed into Fisher's z_k: $z_k = 0.5 \log_e\left(\frac{1+r_k}{1-r_k}\right)$, to ensure that the sampling distribution is approximately normal.

The between-study variance, τ^2 To estimate the random-effects model, the error ζ_k must be considered. To do this, the variance of the distribution of true effect sizes, τ^2, has to be estimated. There are several methods to estimate τ^2, we deploy Maximum Likelihood [19].

The confidence intervals around τ^2 can be estimated using various methods, which depend on the type of τ^2 estimator used. We deploy the *Q-Profile* method [20] which is based on the Q_{gen} statistic: $Q_{gen} = K\sum_{k=1}^{k} w_k^*(\hat{\theta}_k - \hat{\mu})^2$. The Q-Profile method uses an iterative process: $Q_{gen}(\tilde{\tau}^2)$ is calculated repeatedly while increasing the value of τ^2 until the expected value of the lower and upper bound of the confidence interval based on the χ^2 distribution is reached.

Unlike the standard version of Q, which uses the pooled effect based on the fixed-effect model, Qgen is based on the random-effects model and uses the overall effect according to this model, denoted by $\hat{\mu}$, to calculate the deviates. Additionally, Qgen uses weights based on the random-effects model, denoted by w_k^*, in its calculation. The equation for Qgen is given by $Q_{gen} = \sum_{k=1}^{K} w_k^*\left(\hat{\theta}_k - \hat{\mu}\right)^2$, where w_k^* is the random-effects weight $w_k^* = \frac{1}{s_k^2 + \tau^2}$.

The hetereogeneity measure, I^2 We calculate the I^2 statistic [21] to provide an estimate of the magnitude of the between-study heterogeneity. I^2 represents the percentage of the total variability in the effect sizes not due to sampling error, formally expressed as $I^2 = \frac{Q-(K-1)}{Q}$, where K is the total number of studies. Cochran's Q [58] is defined as $Q = \sum_{k=1}^{K} w_k(\hat{\theta}_k - \hat{\theta})^2$. It uses the deviation of each study's observed effect

$\hat{\theta}_k$ from the summary effect $\hat{\theta}$, weighted by the inverse of the study's variance, w_k. The test statistic for Cochran's Q is distributed as chi-squared with $K-1$ degrees of freedom under the null hypothesis of homogeneity. The value of I^2 cannot be lower than 0%. If Q is smaller than $K-1$, 0 is used instead of a negative value.

Prediction intervals Prediction intervals provide a valuable tool for estimating the likely range of effects that future studies may have based on the current evidence. The formula for 95% prediction intervals [26] is calculated as follows:

$\hat{\mu} \pm t_{K-1,0.975}\sqrt{SE_{\hat{\mu}}^2 + \hat{\tau}^2}$ $\quad \hat{\mu} \pm t_{K-1,0.975} SD_{PI}$, where K is the number of studies.

Mixed-effects models The meta-regressions were specified as mixed-effects models: $\hat{\theta}_k = \theta + \beta x_k + \epsilon_k + \zeta_k$. The first error, ϵ_k, represents the sampling error through which a study's effect size deviates from its true effect. The second error, ζ_k, indicates that even the true effect size of a study is only sampled from an overarching distribution of effect sizes.

Intraclass correlation (ICC) We calculated the ICC through a two-way random-effects model (as provided by package `psych`) to assess the reliability of the average of k ratings for each signal i. We describe each rating as $y_{ij} = \mu + r_i + c_j + e_{ij}$, where μ is the average rating, $r_i \sim N(0, \sigma_r^2)$ and $c_j \sim N(0, \sigma_c^2)$ are random effects for the signals and raters, respectively, and e_{ij} is the error term. Then, we compute $\text{ICC} = \frac{\sigma_r^2}{\sigma_r^2 + (\sigma_c^2 + \sigma_\epsilon^2)/k}$ [22].
