# Supplementary material for: Perceived warmth and competence predict callback rates in meta-analyzed North American labor market experiments
Source: PLoS One. 2024 Jul 10;19(7):e0304723. doi: 10.1371/journal.pone.0304723 (PMC11236140; doi:10.1371/journal.pone.0304723)
Supplement: S2 File — (PDF) [file pone.0304723.s002.pdf]

S2 Heterogeneity analysis On our main meta-model, we computed several influence diagnostics (Externally Standardized Residuals, DFFITS Value, Cook's Distance, Covariance Ratio, Leave-One-Out τ^2, Hat Value, Study Weight), which did not nominate any study as an outlier (Fig. S2).

Additionally, we implemented a Graphical display of heterogeneity (GOSH) plot analysis (Fig. S3). For this analysis, we fit all possible subsets 2^{k-1} of our included studies. And plot the pooled effect size against the between-study heterogeneity. Three (k-means, DBSCAN, gmm) clustering algorithms are used to determine patterns. Two (DBSCAN, GMM) algorithms detected the same potential outliers: [28], [59], [60], [34]. Excluding those yields $\theta = .22$, p-value=.19.

Furthermore, we visualized the contribution of each study to the overall heterogeneity against its influence on the pooled effect size (also known as Baujat plot, Fig. S4). [59] showed the highest contribution to heterogeneity, however, its influence on the pooled result was small. Neumark et al. [60] showed a moderate contribution to the overall heterogeneity but a substantial influence on the pooled result.

A leave-one-out robustness analysis also indicated that excluding [60] resulted in the largest decrease in the I^2 statistic, reducing it from 81% to 63.8%. $\theta = .34$, p-value=.026 (Fig. S5).

Influence diagnostics

In the following, we define the measures plotted in Fig. S2. Fig. S2, first panel, displays the *externally standardized residual* of each study, defined as follows:

$$t_k = \frac{\hat{\theta}_k - \hat{\mu}_{\setminus k}}{\sqrt{\mathrm{Var}(\hat{\mu}_{\setminus k}) + \hat{\tau}^2_{\setminus k} + s^2_k}} \tag{1}$$

These residuals are the deviation of each observed effect size $\hat{\theta}_k$ from the pooled effect size. The "external" pooled effect $\hat{\mu}_{\setminus k}$ is obtained by calculating the overall effect without study k. The resulting residual is then standardized by (1) the variance of the external effect $\hat{\mu}_{\setminus k}$), (3) the τ^2 estimate of the external pooled effect, and (3) the variance of k.

Fig. S2, second panel, displays the DFFITS_k. The DFFITS value indicates how much the pooled effect changes when a study k is removed, expressed in standard deviations. Higher values indicate that a study may be influential because its impact on the average effect is larger.

$$DFFITS_k = \frac{\hat{\mu} - \hat{\mu}_{\setminus k}}{\sqrt{\frac{w^*_k}{\sum_{k=1}^{K} w^*_k}(\hat{s}^2_k + \hat{\tau}^2_{\setminus k})}} \tag{2}$$

where w^*_k is the (random-effects) weight of study k.

Fig. S2, third panel displays the Cook's distance value D_k of a study. D_k only takes positive values and is calculated as follows:

$$D_k = \frac{(\hat{\mu} - \hat{\mu}_{\setminus k})^2}{\sqrt{\hat{s}^2_k + \hat{\tau}^2}} \tag{3}$$

Fig. S2, fourth panel displays $\mathrm{CovRatio}_k$. A value below 1 indicates that removing study k results in a more precise estimate of the pooled effect size $\hat{\mu}$.

$$\mathrm{CovRatio}_k = \frac{\mathrm{Var}(\hat{\mu}_{\setminus k})}{\mathrm{Var}(\hat{\mu})} \tag{4}$$

Fig. S2, fifth and sixth panels display Leave-One-Out τ^2 and Q values. The values display the estimated heterogeneity as measured by τ^2 and Cochran's Q if study k is

removed. Lower values of Q, but particularly of τ^2, are desirable since this indicates lower heterogeneity.

Fig. S2, seventh and eigth panels display the study weight and hat value of each study. The hat value is another metric that is equivalent to the study weight.
