# Supplementary material for: Perceived warmth and competence predict callback rates in meta-analyzed North American labor market experiments
Source: PLoS One. 2024 Jul 10;19(7):e0304723. doi: 10.1371/journal.pone.0304723 (PMC11236140; doi:10.1371/journal.pone.0304723)
Supplement: S3 File — (PDF) [file pone.0304723.s003.pdf]

S3 Subgroup analysis: Social perceptions across job types This section looks further into studies that have published their full datasets. In the following analyses, each row corresponds to one CV sent out in the experiment. Columns for each study vary, but in all of them, we have access to the name on the CV and a variety of CV-specific characteristics (e.g., education level, previous experience).

We assume that the explanatory power of social perceptions is dependent on job-specific context. A base model (logit, no classes) would explain callbacks as such:

$$\Pr(\text{Callback} = 1) = \frac{\exp(\alpha + \beta \text{PC1})}{1 + \exp(\alpha + \beta \text{PC1})}$$

Where α is the intercept and β is the coefficient from this estimation. Thus, we compare this model with one where we run interactions with job types. We infer interactions between industries, occupations, and educational levels from six correspondence studies by employing finite mixture models (FMMs) for clustering occupations. FMMs allow multiple latent classes to reveal a more detailed relationship between stereotypes and callbacks across different job types.

FMMs are employed because they allow for the modeling of unobserved heterogeneity by identifying subgroups or clusters within the data. In this context, FMMs are used to identify different classes of occupations that have a similar relationship between callbacks and social perception features.

Mathematically, the FMM can be expressed as:

$$f(y_i) = \sum_{j=1}^{k} \pi_j f_j(y_i)$$

where is the π_j is the mixing proportion for the j-th class, $f(y_i)$ the conditional probability density function for the observed response y_i (callbacks) in the i-th class model, and $k = 2$ is the number of latent classes or clusters. In other words, we estimate a different β for each latent class. The softmax function determines latent class probability:

$$\pi_i = \frac{\exp(\gamma_i)}{\sum_{j=1}^{g} \exp(\gamma_j)} \tag{5}$$

where γ_i is a function of job characteristics. The effect of PC1 on job characteristics was operationalized using text data from job advertisement titles and descriptions and other relevant variables such as industry, occupation, and education level. Industry and occupation variables provided insights into specific sectors and job roles, respectively, while the education level variable captured employers' educational requirements or preferences for certain positions. These additional variables were provided in the published datasets and offered contextual information about the jobs being studied. The dataset was preprocessed and cleaned to eliminate rare industries and occupations, retaining only those that appeared in at least 1% of the observations.

The job description text was parsed, and common words were extracted using the n-gram technique with n=1 (unigrams), which allowed for identifying meaningful patterns in the text data [61]. Unigrams are single words from the text, which help capture the frequency of individual words and their potential importance in characterizing jobs.

For each study, we fit a base model with no latent classes and compare it to separate FMMs with two classes. We then evaluate the fit of these models by comparing their Bayesian information criterion (BIC) values to determine the best model for each study. We then use the best model to analyze the potential differences between the two classes.

Table S9 shows that, for all studies, the FMM model had lower BICs than the base model.

After fitting the finite mixture models, we obtain the predicted posterior probabilities for each class. These probabilities indicate the likelihood of each observation (i.e., CV) belonging to a specific class. We choose the one with the highest predicted posterior probability to assign a class to each observation. This approach ensures that each CV is assigned to the class with the highest probability of belonging, maximizing the model's overall fit.

With the CVs assigned to their respective classes, we then compute the correlation coefficients between PC1 and callbacks separately for each class. This allows us to investigate how the relationship between social perceptions and callbacks differs across job types or classes. By examining these correlations, we can better understand the role of social perceptions in driving callback rates for different types of jobs and determine if specific job characteristics are more or less sensitive to social perceptions.

We then compare the job characteristics between classes across these studies. Table S10 presents the correlation coefficients between PC1 and callbacks for each study under three different scenarios: the base model without latent classes, within Class 1, and Class 2. For instance, in [2], the correlation between PC1 and callbacks is 0.05 in the base model but increases to 0.57 within Class 1. However, no correlation coefficient is reported for Class 2 in [2], as indicated by "NA" in the table. This implies that there is no variation in callbacks within this class—either all CVs received callbacks (all 1s) or none of them did (all 0s).

Similarly, we can observe varying correlation coefficients within each class compared to the base model for the other studies. These results highlight that the relationship between social perceptions and callbacks is not uniform across different job types and that some jobs might exhibit stronger or weaker associations between social perceptions and callbacks than others.

For the following analyses, we will focus only on Farber and Nunley, as these studies produced classes with enough callback variation for us to draw meaningful conclusions. Table S11 presents the text data from job titles and descriptions and other relevant variables with the largest difference between classes. This comparison allows us to understand the qualitative differences between the two better.

From the table, we can observe noticeable differences between the two classes regarding job characteristics. In the Nunley dataset, Class 1 has a higher prevalence of Manager, Analyst, Management, Finance, and Specialist positions, while Representative, Insurance, Entry Level, and Sales roles dominate Class 2. This suggests that Class 1 might comprise more advanced, specialized, or managerial positions, whereas Class 2 consists of more entry-level or sales-oriented roles.

In the Farber dataset, Class 1 is characterized by a higher presence of Health Care and Social Assistance, Professional, Scientific, and Technical Services, Finance and Insurance, Retail Trade, and Manufacturing industries. In contrast, Class 2 is more heavily represented in Arts, Entertainment, and Recreation, Educational Services, Repair and Maintenance, and Real Estate and Rental and Leasing industries. Additionally, the Administrator occupation is more common in Class 1, while Class 2 shows a more diverse range of industries, focusing on non-managerial positions. This indicates that Class 1 might be associated with more professional or technical industries. In contrast, Class 2 is linked to a broader variety of roles, primarily in service-oriented and less specialized sectors.

With these qualitative differences, we can analyze the differences in the correlation between callbacks and the first principal component (PC1) for each study and class. We used a weighted Pearson correlation coefficient calculated through the 'cov.wt' function from the R 'stats' package, with weights proportional to the number of resumes sent

with each name within a category. To assess the statistical significance of these correlations, we implemented a robust permutation-based method called Perm-Z2 ([31]). This approach addresses issues related to small sample sizes and deviations from normality. By employing this studentized permutation test, we can achieve more accurate type I error control and statistical inference, making Perm-Z2 particularly suitable for our context [31,32].

In [33], the correlation between callbacks and PC1 (representing positive social perceptions, including warmth and competence) is higher in Class 2 (0.26) than in Class 1 (0.17). Class 1 contains job titles associated with more professional and technical roles, while Class 2 comprises a more diverse range of service-oriented and less specialized roles. This suggests that the broader variety of roles in Class 2 might benefit more from positive social perceptions, especially warmth, when it comes to receiving callbacks.

In contrast, [33] shows a slightly higher correlation between callbacks and PC1 in Class 1 (0.80) than in Class 2 (0.77). Class 1 in this study is characterized by advanced, specialized, or managerial positions, while Class 2 includes entry-level or sales-oriented roles. These findings indicate that competence and warmth are highly valued in both classes but may be slightly more important for receiving callbacks in Class 1, which consists of more specialized positions.

However, to compare the correlation coefficients across classes, we need to use the Fisher transformation due to the highly skewed distribution of correlation coefficients in [34]. The Fisher transformation is given by:

$$r' = \frac{1}{2} \ln \frac{1+r}{1-r}$$

After applying the Fisher transformation and calculating the z-values for the difference between correlation coefficients using the formula:

$$z = \frac{r'_1 - r'_2}{\sqrt{\frac{1}{n_1-3} + \frac{1}{n_2-3}}}$$

We find that for the Farber study, $z = 0.200$, and for Nunley, $z = 0.124$.

The magnitudes of the z-scores indicate the difference size between the two classes' correlation coefficients. In this case, neither the Farber study's z-score (0.200) nor the Nunley study's z-score (0.124) exceed the critical value, indicating that the differences in correlation coefficients between the classes are not statistically significant at the $p < 0.05$ level.

It is important to note that PC1 represents warmth and competence, and this amalgamation of warmth and competence makes it difficult to distinguish the specific role each dimension plays, particularly in entry-level positions where the contribution of warmth and competence to callbacks may differ.

We conducted an exploratory analysis using partial correlations to better understand the interplay between warmth and competence in influencing callbacks for various job positions. Partial correlations allow us to examine the relationship between two variables while controlling for the influence of one or more other variables. We assessed the relationships between warmth and competence scores separately, controlling for the other dimension, as seen in Table S12.

These results suggest that different job categories may have distinct relationships between warmth and competence. In Farber's study, warmth appears to be positively related to callbacks in both job classes, while competence is negatively related. However, the magnitude of the relationship between warmth and callbacks is greater in Class 2, which includes jobs requiring more social interaction, aligning with our previous qualitative analysis. The negative relationship between competence and callbacks is

stronger in Class 1, suggesting that competence may not be as crucial for jobs that involve more routine tasks and lower technical skills.

In contrast, Nunley's study shows a mixed relationship for warmth, with a negative relationship in Class 1 and a positive one in Class 2 and a positive relationship between competence and callbacks in both classes. The negative relationship between warmth and callbacks in Class 1 is larger in magnitude than the positive relationship in Class 2, indicating that warmth may be less important or even detrimental for jobs requiring higher cognitive and technical skills. Conversely, the positive relationship between competence and callbacks is notably stronger in Class 1, which consists of jobs requiring higher cognitive and technical skills, highlighting the importance of competence in these positions.

It is important to note that this analysis is exploratory, and further research is certainly needed to validate these findings. However, these results do provide preliminary evidence that the relationships between warmth, competence, and job callbacks may be more complex than initially thought.

Table S1. Published studies for which raw data was obtained. The numbers represent the count of signals (names) per race, gender, and study.

gender	black	white
[63]		
female	9	9
male	9	9
[64]		
female	–	12
[28]		
female	5	5
[65]		
female	16	17
male	18	17
[66]		
female	–	313
male	–	218
[67]		
female	2	2
male	2	2
[68]		
female	–	8
male	–	8
[69]		
male	–	6

Note: Neumark, Bertrand, Farber, and Kline varied the first name only. Oreopoulos, Flake, Leasure, Widner, and Jacquemet varied the first and the last name.

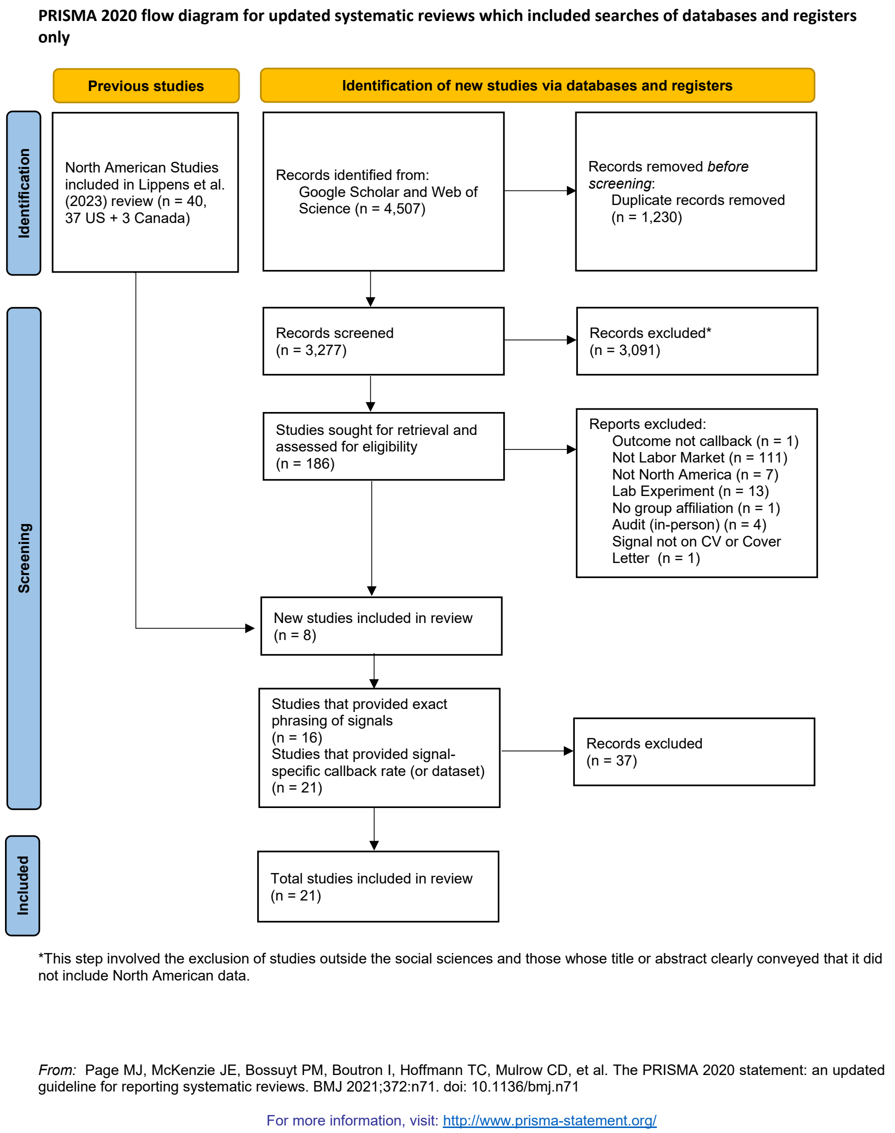

Fig S1. Prisma chart.

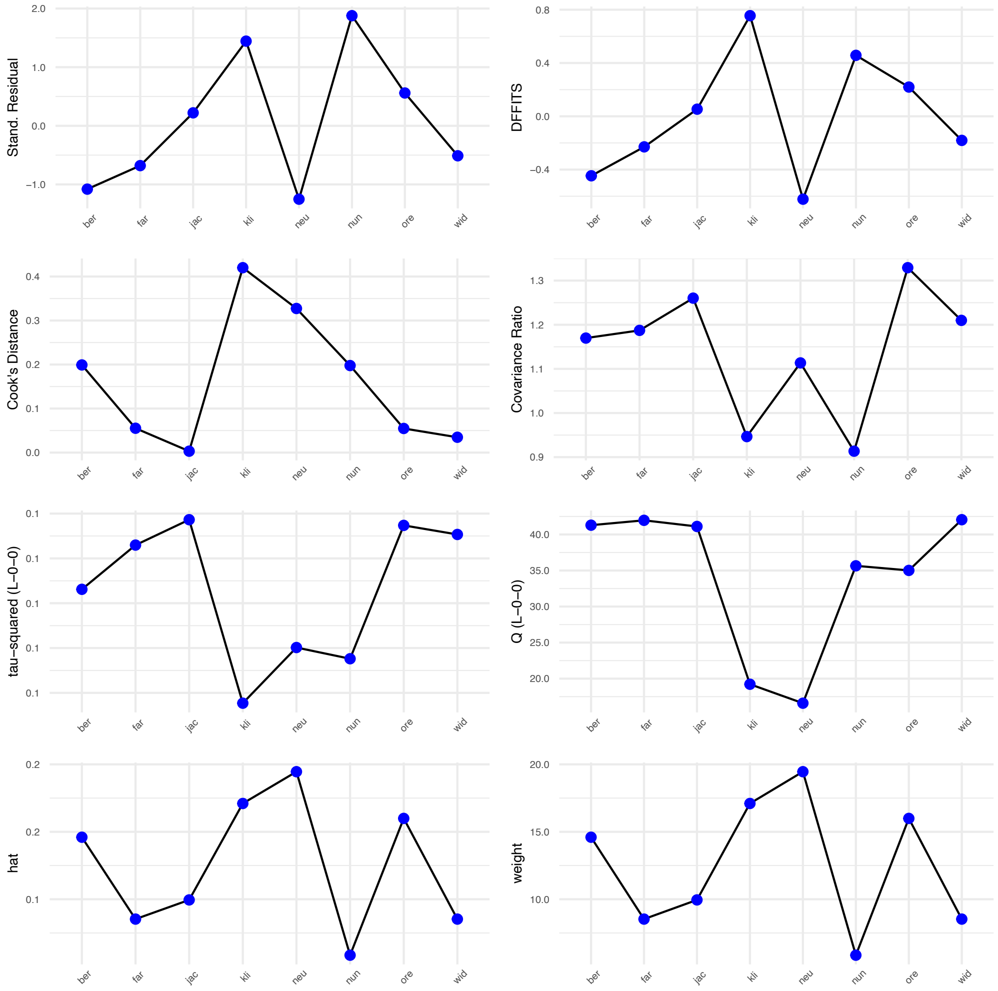

Fig S2. Different influence measures for each study. Those measures help to identify potential outliers that do not fit well into the meta-analysis model. No study was detected as an outlier based on these measures.

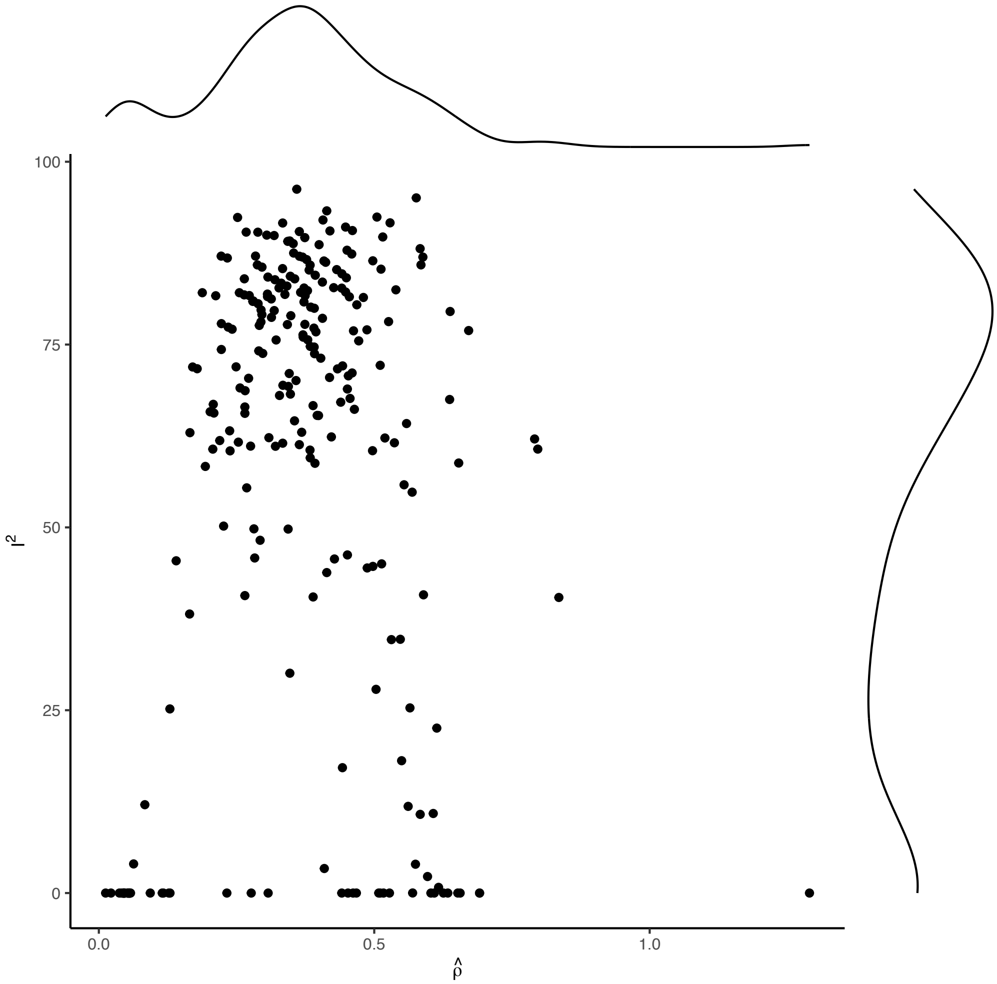

Fig S3. GOSH Plot. We implemented a Graphical display of heterogeneity (GOSH) plot analysis. For this analysis, we fit all possible subsets 2^{k-1} of our k included studies. Each subset's pool effect size $\hat{\rho}$ is plotted on the x-axis, and the between-study heterogeneity I^2 on the y-axis. Three (k-means, DBSCAN, gmm) clustering algorithms are used to determine patterns in the above scatter plot. The three algorithms did not consistently identify clusters therefore, we conclude that based on this analysis, no single study needs to be excluded from estimating the meta-model.

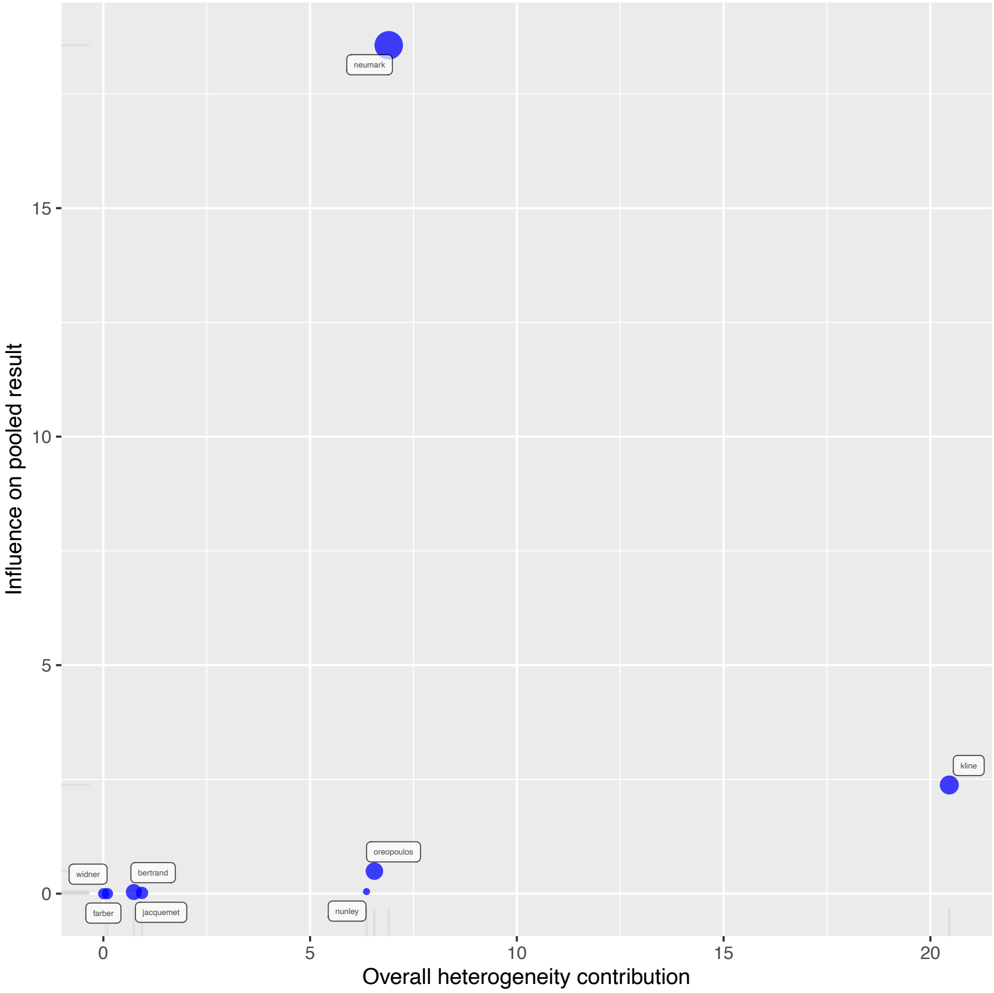

Fig S4. Baujat plot. The Baujat plot [62] is a diagnostic plot used to identify studies that disproportionately contribute to heterogeneity in a meta-analysis. The plot displays the contribution of each study to the overall heterogeneity (measured by Cochran's Q) on the x-axis and its impact on the pooled effect size (defined as the standardized squared difference between the overall estimate based on an equal-effects model with and without the i$^t h$ study included in the model) on the y-axis. Our analysis found that [59] significantly influenced the heterogeneity but did not significantly affect the pooled effect size. On the other hand, [60] contributed moderately to the overall heterogeneity but substantially impacted the pooled effect size.

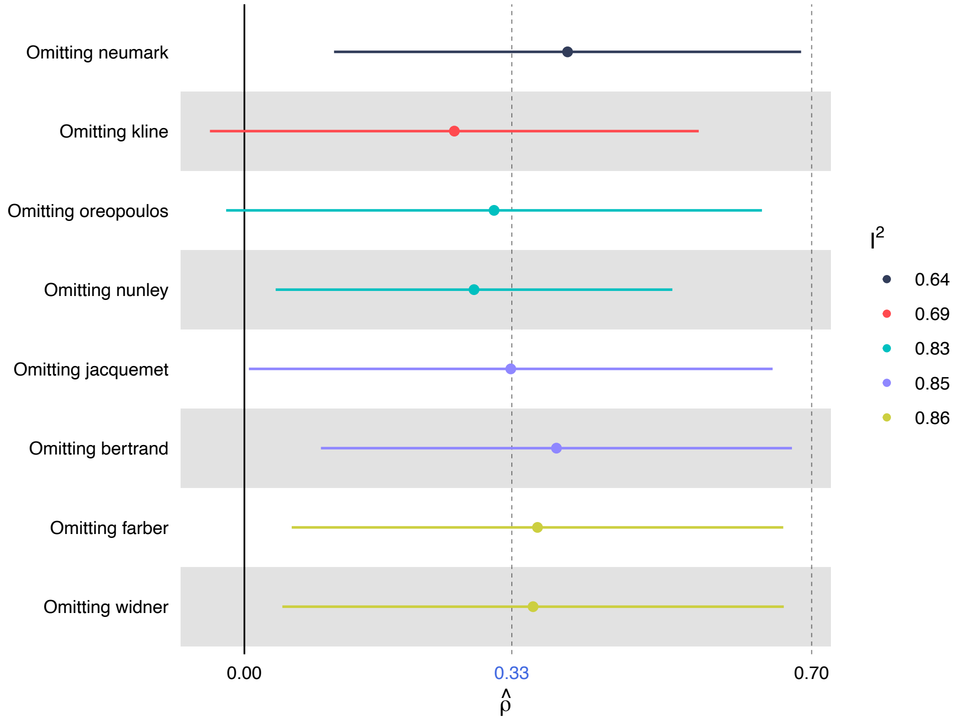

Fig S5. Forest plot. The plot displays the overall effect and I^2 heterogeneity of all meta-analyses with ρ(callback, PC1) as effectsize that were conducted using the leave-one-out method. The forest plot is sorted by the I^2 value of the leave-one-out meta-analyses. The results show that excluding [60] leads to the largest reduction in I^2, reducing it from 82% to 64%.

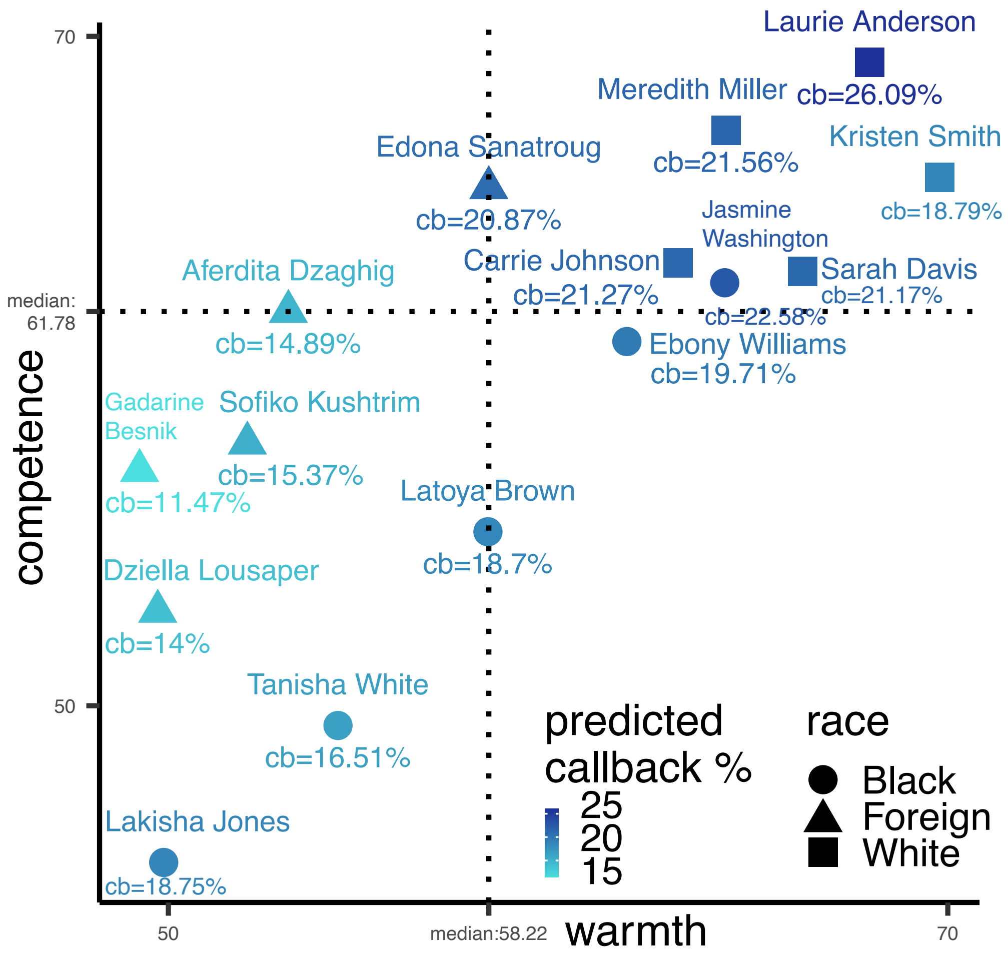

Fig S6. Warmth and competence ratings for names in [28]. Each dot's shape represents the name's race, either black, white, or foreign-sounding. The color of the dots corresponds to the predicted callback. The predictions were generated using a linear model of PC1 on callback, with the training set consisting of all names except one. The predicted callback values for each name are displayed beneath the corresponding dot.

Table S2. ICC values for names

	Warmth		Competence		Mean	
	ICC	score	ICC	score	ICC	score
[28]	0.98	excellent	0.97	excellent	0.97	excellent
[59]	0.95	excellent	0.96	excellent	0.95	excellent
[69]	0.97	excellent	0.99	excellent	0.98	excellent
[2]	0.83	good	0.69	moderate	0.76	good
[60]	0.94	excellent	0.81	good	0.87	good
[34]	0.65	moderate	0.93	excellent	0.79	good
[70]	0.91	excellent	0.89	good	0.90	good
[33]	0.86	good	0.50	poor	0.68	moderate

Note: Average score intraclass correlations (ICCs) were used as an index of interrater reliability of warmth competence ratings. A twoway model with random effects for raters and subjects (amount of levels in category) was used. Between rater agreement was estimated. The unit of analysis was averages. Column "Mean" presents the average warmth and competence ICC score.

Table S3. ICC values for categories

		Warmth		Competence		Mean	
	category	ICC	score	ICC	score	ICC	score
[71]	health	0.96	excellent	0.93	excellent	0.95	excellent
[72]	health	0.96	excellent	0.97	excellent	0.97	excellent
[73]	parenthood	0.97	excellent	0.92	excellent	0.95	excellent
[74]	unemployed	0.96	excellent	0.98	excellent	0.97	excellent
[75]	religion	0.95	excellent	0.94	excellent	0.95	excellent
[76]	nationality	0.91	excellent	0.93	excellent	0.92	excellent
[77]	sexuality	0.83	good	0.94	excellent	0.89	good
[33]	age	0.55	moderate	0.79	good	0.67	moderate
[78]	wealth	0.82	good	0.63	moderate	0.72	moderate
[79]	sexuality	0.64	moderate	0.51	moderate	0.58	moderate
[80]	sexuality	0.04	poor	0.95	excellent	0.50	poor
[81]	parenthood	0.87	good	0.00	poor	0.43	poor
[82]	military	0.00	poor	0.00	poor	0.00	poor
[59]	sexuality	0.00	poor	0.94	excellent	0.47	poor
[60]	age	0.08	poor	0.33	poor	0.21	poor
[83]	wealth	0.00	poor	0.97	excellent	0.49	poor

Note: Average score intraclass correlations (ICCs) were used as an index of interrater reliability of warmth competence ratings. A twoway model with random effects for raters and subjects (amount of levels in category) was used. Between rater agreement was estimated. The unit of analysis was averages. Column "Mean" presents the average warmth and competence ICC score.

Table S4. Results of a random effects model with ρ(warmth, competence) for names

		95% CI			
	ρ	lower	upper	p-value	SE
by study					
[2]	0.616	0.378	1.060	0.000	0.174
[33]	0.408	-0.220	1.086	0.194	0.333
[84]	0.900	1.241	1.700	0.000	0.117
[85]	0.826	0.585	1.767	0.000	0.302
[28]	0.845	0.715	1.763	0.000	0.267
[59]	0.900	1.241	1.700	0.000	0.117
[60]	0.631	0.565	0.923	0.000	0.091
[34]	0.740	0.073	1.826	0.034	0.447
[70]	0.565	0.334	0.946	0.000	0.156
[69]	0.915	0.904	2.210	0.000	0.333
pooled $\hat{\rho}$	0.780	0.759	1.330	0.000	0.126

Note: Random effects model of 10 studies with 418 observations using inverse variance method. Restricted maximum-likelihood estimator for τ^2 and Hartung-Knapp adjustment (df = 8). Confidence intervals for τ^2 and τ were estimated using the Q-Profile method. Fisher's z transformation was used for correlations.

Table S5. Results of a random effects model with ρ(warmth, competence) for categories

		95% CI			
	ρ	lower	upper	p-value	SE
by study					
[71]	0.574	0.514	0.794	0	0.072
[80]	0.546	0.474	0.752	0	0.071
[81]	0.645	0.626	0.907	0	0.072
[33]	0.602	0.616	0.778	0	0.041
[82]	0.342	0.216	0.496	0	0.072
[72]	0.699	0.724	1.005	0	0.072
[73]	0.710	0.749	1.026	0	0.071
[59]	0.416	0.304	0.582	0	0.071
[77]	0.456	0.394	0.591	0	0.050
[74]	0.776	0.922	1.149	0	0.058
[60]	0.724	0.802	1.030	0	0.058
[78]	0.583	0.526	0.808	0	0.072
[83]	0.425	0.313	0.594	0	0.072
[79]	0.437	0.327	0.609	0	0.072
[75]	0.696	0.789	0.929	0	0.036
[76]	0.639	0.724	0.791	0	0.017
pooled $\hat{\rho}$	0.595	0.579	0.792	0	0.050

Note: Random effects model of 16 studies with 7830 observations using inverse variance method. Restricted maximum-likelihood estimator for τ^2 and Hartung-Knapp adjustment (df = 15). Confidence intervals for τ^2 and τ were estimated using the Q-Profile method. Fisher's z transformation was used for correlations.

Table S6. Pooling effect sizes competence, warmth, and callback for the categories race and gender

	estimate	95% CI lower	95% CI upper	p-value	SE
competence[1]					
black[2]	-11.52	-23.74	0.71	0.06	3.84
female[3]	-3.07	-9.56	3.42	0.32	2.91
warmth[1]					
black[2]	-6.72	-19.19	5.76	0.19	3.92
female[3]	2.88	-4.39	10.16	0.40	3.27
callback[4]					
black[5]	0.79	-0.51	0.04	0.07	0.09
female[6]	1.02	-0.03	0.06	0.36	0.01

Note: [1]Statistic is a warmth/competence rating expressed on a scale from 0 to 100. Models involve the inverse variance method and a restricted maximum-likelihood estimator for τ^2. The Q-Profile method was used to compute the confidence interval of τ^2 and τ, and a Hartung-Knapp (HK) adjustment was applied for the random effects model, with degrees of freedom set to 10.
[2]k=4 studies, o=687 observations.
[3]k=11 studies, o=816 observations.
[4]The effect size represents a risk ratio. The Mantel-Haenszel method was used to calculate the overall effect size, with the Paule-Mandel estimator used to estimate the between-study variance τ^2. A random-effects model was employed with the Hartung-Knapp (HK) adjustment to account for potential bias due to small sample sizes. The model had 1 degree of freedom (df = 1).
[5]k=4 studies, o=89872 observations.
[6]k=4 studies, o=143860 observations.

Table S7. Estimates of linear models of PC1 on callback by category

	$\hat{\beta}$	SE	statistic	p-value
wealth				
[78]	-0.84			
[83]	0.00			
unemployed				
[74]	0.02	0.01	1.74	0.33
sexuality				
[80]	-0.10			
[59]	0.03			
[77]	0.05	0.23	0.22	0.85
[79]	-0.82			
parenthood				
[81]	-0.03			
[73]	0.07			
nationality				
[76]	0.04	0.02	1.52	0.14
military				
[82]	1.41			
health				
[71]	0.00			
[72]	0.07			
age				
[33]	0.04	0.02	1.63	0.18
[60]	0.20	0.23	0.87	0.54
[75]	0.01	0.00	1.68	0.14

Note: We used the available data points to fit a linear model for each category, although many categories only had two data points. Empty cells indicate that the relevant statistics could not be computed. We focus exclusively on the slope. It should be noted that the data from published literature is limited, with only a few studies per category and a few levels per category. Our main goal with this analysis is to provide a preliminary glimpse of the trend. We found a positive association between PC1 and callback for some categories, but we found mixed evidence for others. For example, the sexuality category had two studies with a negative slope and two with a positive slope. Empty cells indicate that there were not enough data points to estimate the relevant statistics.

Table S8. Mixed effects models with varying independent variables

	$\hat{\beta}$	SE	p-value	95% CI lower	95% CI upper	R^2
callback ∼ race						
intrcpt	−2.07	0.30	0.00	−2.67	−1.48	0.95
Black	0.71	0.79	0.37	−0.85	2.27	0.95
callback ∼ PC1						
intrcpt	−1.98	0.25	0.00	−2.48	−1.48	4.36
PC1	0.99	0.30	0.00	0.41	1.57	4.36
callback ∼ race + PC1						
intrcpt	−2.14	0.30	0.00	−2.73	−1.56	6.13
PC1	1.07	0.32	0.00	0.45	1.69	6.13
Black	1.19	0.79	0.13	−0.36	2.74	6.13

Note: Mixed-Effects Models (k = 644; τ^2 estimator: ML)

Our findings suggest that PC1 is a positive and significant predictor of callback. The table investigates how PC1 compares as a predictor to categorical variables that are commonly used in correspondence studies. To this end, we fit three mixed-effects models, of different predictors (PC1, race, PC1+race) on callback. Our analysis reveals that the R^2 value is highest for model three, as expected. However, we also observe that the R^2 value is substantially higher (4.36) for model PC1 compared to the model with race only (.95). Notably, our results show that race is never a significant predictor of callback in either model one or model three, whereas PC1 is a significant predictor in both models. These findings underscore the importance of social perception as a valuable predictor of callback (Table S8).

Table S9. Comparison of BIC values for base (logit, no classes) and FMM models in each study

	N	df	BIC
[33]			
Base	8899	2	6227.6
FMM	8665	20	6160.1
[70]			
Base	12910	2	8351.3
FMM	12910	26	8302.0
[60]			
Base	31523	2	27007.7
FMM	31523	8	26172.0
[34]			
Base	9396	2	8463.0
FMM	9396	59	7613.8
[59]			
Base	74946	2	82759.8
FMM	68297	22	74504.6
[2]			
Base	5635	2	3135.5
FMM	5635	10	3118.5

Table S10. Correlation coefficients r(PC1, callbacks) in the base model (logit, no classes) and within each class for each study

	Base (no class)		Class 1		Class 2	
Study	r (base)	N	r	Proportion (%)	r	Proportion (%)
[33]	0.07 ($p = .400$)	9,240	0.17 ($p = .340$)	64.92	0.26 ($p = .201$)	35.08
[34]	0.86 ($p = .006$)	9,396	0.80 ($p = .009$)	23.88	0.77 ($p = .022$)	76.12
[2]	0.05 ($p = .516$)	5,635	0.57 ($p < .001$)	98.76	NA	1.24
[59]	0.60 ($p < .001$)	74,946	NA	22.67	0.20 ($p = .056$)	77.33
[70]	0.49 ($p < .001$)	12,910	-0.61 ($p = .750$)	90.08	NA	9.92

Table S11. Mean differences in job characteristics between classes for [34] and [33]

Variable	Mean (Class 1)	SE (Class 1)	Mean (Class 2)	SE (Class 2)	Z score	p-value
[34]						
Manager	0.243	0.005	0.129	0.007	11.68	1.63E-31
Analyst	0.104	0.004	0.000	0.000	16.22	3.82E-59
Management	0.202	0.005	0.101	0.006	11.04	2.36E-28
Finance	0.195	0.005	0.111	0.006	9.33	1.03E-20
Specialist	0.078	0.003	0.003	0.001	13.16	1.45E-39
Representative	0.097	0.004	0.225	0.009	-15.94	3.46E-57
Insurance	0.086	0.003	0.233	0.009	-18.78	1.08E-78
Level	0.005	0.001	0.200	0.008	-36.23	1.9E-287
Entry	0.003	0.001	0.201	0.008	-37.43	1.2E-306
Sales	0.194	0.005	0.562	0.010	-34.14	1.9E-255
[33]						
Health Care and Social Assistance	0.260	0.006	0.074	0.005	20.77	7.3E-96
Professional, Scientific, and Technical Services	0.240	0.006	0.079	0.005	18.40	1.2E-75
Finance and Insurance	0.099	0.004	0.009	0.002	15.84	1.72E-56
Retail Trade	0.103	0.004	0.037	0.003	10.79	3.89E-27
Manufacturing	0.069	0.003	0.003	0.001	13.85	1.2E-43
Administrator	0.477	0.006	0.549	0.009	-6.65	2.96E-11
Arts, Entertainment, and Recreation	0.000	0.000	0.105	0.006	-25.60	1.4E-144
Educational Services	0.004	0.001	0.113	0.006	-25.00	5.9E-138
Repair and Maintenance	0.000	0.000	0.175	0.007	-33.37	3.4E-244
Real Estate and Rental and Leasing	0.000	0.000	0.215	0.008	-37.26	6.7E-304

Table S12. Partial Correlation coefficients between warmth, competence, and callbacks within each class (i.e., the estimate of the correlation between warmth and callbacks, controlling for other competence (and vice-versa).

	Partial Correlation	
	Warmth	Competence
[33]		
Class 1	0.26 (p = .188)	-0.20 (p = .281)
Class 2	0.35 (p = .142)	-0.15 (p = .302)
[34]		
Class 1	-0.34 (p = .200)	0.83 (p = .022)
Class 2	0.28 (p = .320)	0.48 (p = .213)

Table S13. Published studies from which categories were extracted

Reference	Category
[71]	Health
[80]	Sexuality
[81]	Parenthood
[33]	Age
[82]	Military
[72]	Health
[73]	Parenthood
[59]	Sexuality
[77]	Sexuality
[74]	Unemployed
[60]	Age
[78]	Wealth
[83]	Wealth
[79]	Sexuality
[75]	Religion
[76]	Nationality
