# Supplementary material for: Perceived warmth and competence predict callback rates in meta-analyzed North American labor market experiments
Source: PLoS One. 2024 Jul 10;19(7):e0304723. doi: 10.1371/journal.pone.0304723 (PMC11236140; doi:10.1371/journal.pone.0304723)
Supplement: S4 File — (PDF) [file pone.0304723.s004.pdf]

S4: Prolific sample Our sample size was based on precedent from our past data collections [13] in which we have found that measures of warmth and competence become stable at around 25 ratings per target per attribute. Overall, people show remarkable agreement in their perceptions of these kinds of social groups in studies other than ours. For example, [13], which collected 50 ratings per target (person) per attribute per population reported that the correlation between ratings of targets by (i) mTurk participants and (ii) Berkeley undergraduates was .98 (pearson's r). In [86], the correlations between ratings of targets by (i) mTurk participants and (ii) fMRI study participants in Virginia were .94 (warmth) and .98 (competence). Finally, in our past research by some of us using 50 ratings per target per attribute, we were able to predict outcomes of field studies with good accuracy [13], further supporting the idea that this number of ratings gave meaningful estimates. To corroborate the findings of previous literature with our own data, we conducted a point of stability estimation as outlined by [87]. This sequential sampling method assesses the number of observations required before additional data would not significantly alter the mean value. The process involves defining a corridor of stability (COS), where 95% of the sampled mean ratings (in our case, for warmth and competence) are expected to reside. The smallest sample size n meeting this criterion is termed the point of stability (POS). Selecting the COS is contingent on the rating scale used, a decision that must be made by the researcher. Hehman et al. (2018) selected a COS of +/-1 for a 7-point Likert scale. In contrast, we opted for a COS of +/-5 on a 100-point scale, a considerably more conservative approach. Our findings indicate that for both warmth and competence ratings, the resulting POS is lower than the average number of raters we gathered per category level (99.1 raters) or name (85.9 raters).

Sample Characterisitcs: The Prolific participants constituted a non-convenience, compensated sample with a requisite North American cultural background. This criterion was pivotal, as shared cultural backgrounds are known to foster similar stereotype perceptions, ensuring the recruited sample's stereotypes aligned with those of the study's hiring decision-makers. Post-rating, participants were queried on demographics (race, gender, age) and quality controls (e.g., native language proficiency).

Specifically, for the sample rating names: 57.52% identified as female, with an average age of 37.62 years. The predominant ethnicity was White/Caucasian (62.38%), and the most common education level was a bachelor's degree (33.91%). A majority were in stable employment (52.1%), with the most reported income range being $25,000 to $49,999 (26.6%). A significant 97.5% were native speakers. For the sample rating categories: 50.1% identified as female, with 39.1% in the 25–34 age bracket. A larger majority were White/Caucasian (77.7%), and 31.2% held a bachelor's degree.

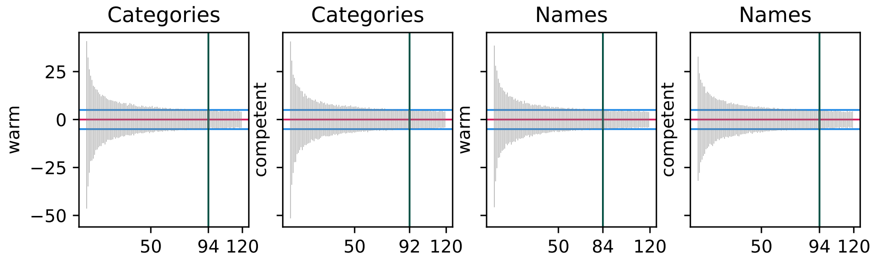

Fig S7. Point of stability (POS) plots for 'warm' and 'competent' ratings. Vertical grey lines indicate 95% of the rating averages at each sample size (n). The blue horizontal lines denote the corridor of stability (COS) at +/- 5. The vertical green lines mark the POS for each rating. Across all four panels, the POS ranges from 84–94, which closely aligns with the average numbers of raters collected per signal in our study, which were 85.9 per name and 99.1 by category level.

Employment was stable for 50.5%, with office-focused roles accounting for 36.6%. The income range of $25,000 to $49,999 was most common (27.2%). A notable 77.2% identified as agnostic, atheist, or non-religious. Lack of resume review experience was reported by 66.3%, and a small fraction (4.95%) had current or past military service.

Choice: Our choice to use a Prolific sample is grounded in literature suggesting that stereotypes are (i) influenced by cultural backgrounds and (ii) pervasively shared within a culture. Consequently, individuals with similar cultural backgrounds are likely to hold comparable stereotypes, regardless of their professional background. This assumption holds even when considering participants from varied professions, as our inquiry does not investigate industry-specific perceptions but rather aims to understand societal views on a particular social signal. While one might argue that recruiters, owing to their training, could be less prone to stereotypical assessments in hiring contexts, the question we posed in the online survey transcends specific industries and focuses on broader societal perceptions. The specific wording was: "In your opinion, what does the average American think about this person? Even if you disagree. [signal, e.g. name]" Therefore, we do not anticipate significant variations in responses across samples drawn from different professional backgrounds.

Furthermore, leveraging stereotypes from one sample to predict behaviors in another offers a conservative approach to evaluating the impact of stereotypes on actions. This method likely leads to an underestimation of the effect size compared to directly measuring decision-makers' stereotypes. Our primary concern is with the influence of broad cultural stereotypes on decision-making processes. The extent to which individuals' actions reflect their personal stereotypes, which may not align with societal norms, represents a separate and potentially less consequential issue. This is because societal-level disparities arise when collective decision-making is guided by uniform assumptions based on shared stereotypes.
