# Supplementary material for: Perceived warmth and competence predict callback rates in meta-analyzed North American labor market experiments
Source: PLoS One. 2024 Jul 10;19(7):e0304723. doi: 10.1371/journal.pone.0304723 (PMC11236140; doi:10.1371/journal.pone.0304723)
Supplement: Attachment — Submitted filename: response_PLOS.pdf [file pone.0304723.s005.pdf]

March 15, 2024

Thank you for your positive feedback and constructive criticisms. We have addressed all the points raised below and sincerely believe these revisions have significantly improved the manuscript.

---

1. From the methods section, it is unclear what the authors' justification is for using a random-effects model meta-analysis. I think it would be valuable to include a short (1-3 sentences) argument for why this model is the appropriate one to use. For instance, did all papers only include one study or did some include multiple? Did several papers originate from the same set of authors/universities/labs? Basically, are there any specific clusters in the data that would have made a mixed-effect model meta-analysis a more appropriate choice?

The following rationale was added to the subsection of manuscript on Statistical Analysis:

> We chose a random-effects model for our analysis due to the inherent heterogeneity across studies. A random-effects model is suitable for our varied dataset as it accounts for differences between studies beyond sampling error, unlike a fixed-effects model. Additionally, since each study contributed a single effect size in our analysis of names, a mixed-effects model would not offer additional analytical benefits.

---

2. What is the rationale for the sample size of the Prolific raters? Also, was this a convenience (vs. nationally representative) Prolific sample and how was the sample quality-checked? Were there for instance any attention/ bot/ comprehension/ quality- checks? Since the ICC's are also an integral part of assessing the quality of the rating for the raters, I suggest the authors draw these results more to the front in the method section on p. 5.

We include detailed information as Supplementary Information 4 (SI4) in the paper.

> **Sample size**: Our sample size was based on precedent from our past data collections [10] in which we have found that measures of warmth and competence become stable at around 25 ratings per target per attribute. Overall, people show remarkable agreement in their perceptions of these kinds of social groups in studies other than ours. For example, [10], which collected 50 ratings per target (person) per attribute per population reported that the correlation between ratings of targets by (i) mTurk participants and (ii) Berkeley undergraduates was .98 (pearson's r). In [8], the correlations between ratings of targets by (i) mTurk participants and (ii) fMRI study participants in Virginia were .94 (warmth) and .98 (competence). Finally, in our past research by some of us using 50 ratings per target per attribute, we were able to predict outcomes of field studies with good accuracy [10], further supporting the idea that this number of ratings gave meaningful estimates. To corroborate the findings of previous literature with our own data, we conducted a point of stability estimation as outlined by [9]. This sequential sampling method assesses the number of observations required before additional data would not significantly alter the mean value. The process involves defining a corridor of stability (COS), where 95% of the sampled mean ratings (in our case, for warmth and competence) are expected to reside. The smallest sample size $n$ meeting this criterion is termed the point of stability (POS). Selecting the COS is contingent on the rating scale used, a decision that must be made by the researcher. Hehman et al. (2018) selected a COS of +/-1 for a 7-point Likert scale. In contrast, we opted for a COS of +/-5 on a 100-point scale, a considerably more conservative approach. Our findings indicate that for both warmth and competence ratings, the resulting POS is lower than the average number of raters we gathered per category level (99.1 raters) or name (85.9 raters).
>
> **Sample Characterisitcs:** The Prolific participants constituted a non-convenience, compensated sample with a requisite North American cultural background. This criterion was pivotal, as shared cultural backgrounds are known to foster similar stereotype perceptions, ensuring the recruited sample's stereotypes aligned with those of the study's hiring decision-makers. Post-rating, participants were queried on demographics (race, gender, age) and quality controls (e.g., native language proficiency).

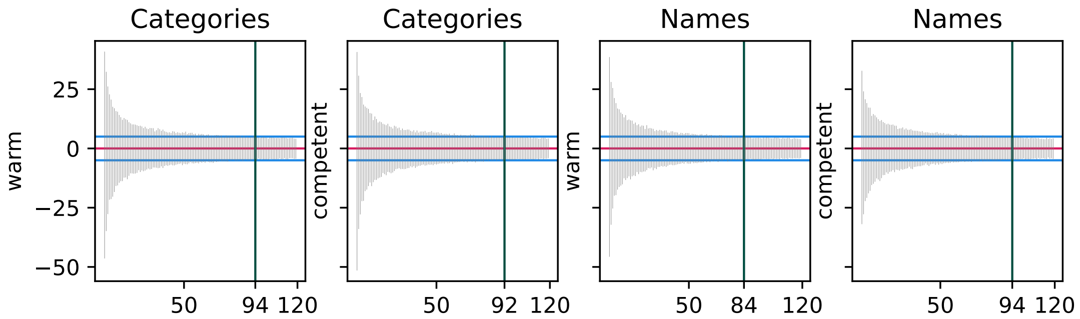


Figure 1: Point of stability (POS) plots for 'warm' and 'competent' ratings. Vertical grey lines indicate 95% of the rating averages at each sample size ($n$). The blue horizontal lines denote the corridor of stability (COS) at +/- 5. The vertical green lines mark the POS for each rating. Across all four panels, the POS ranges from 84–94, which closely aligns with the average numbers of raters collected per signal in our study, which were 85.9 per name and 99.1 by category level.

> Specifically, for the sample rating names: 57.52% identified as female, with an average age of 37.62 years. The predominant ethnicity was White/Caucasian (62.38%), and the most common education level was a bachelor's degree (33.91%). A majority were in stable employment (52.1%), with the most reported income range being $25,000 to $49,999 (26.6%). A significant 97.5% were native speakers. For the sample rating categories: 50.1% identified as female, with 39.1% in the 25–34 age bracket. A larger majority were White/Caucasian (77.7%), and 31.2% held a bachelor's degree. Employment was stable for 50.5%, with office-focused roles accounting for 36.6%. The income range of $25,000 to $49,999 was most common (27.2%). A notable 77.2% identified as agnostic, atheist, or non-religious. Lack of resume review experience was reported by 66.3%, and a small fraction (4.95%) had current or past military service.

Furthermore, we reference SI4 and provide the most important information as a new subsection in Materials and Methods:

> We aim to predict callback rates based on perceived stereotypes. Recognizing that stereotypes are shaped by cultural contexts, we selected raters with North American backgrounds to match the cultural perspectives of recruiters in North American labor market experiments. Participants were recruited through Prolific to provide warmth and competence ratings, with a total of 787 raters for both names (averaging 85.9 per name) and categories (averaging 99.1 per category level). The number of participants to recruit was guided by literature [10] and a point of stability estimation, indicating that mean ratings would stabilize with no significant changes beyond approximately 90 participants (detailed sample size information in SI **??**).
>
> Following the rating process, participants provided demographic information. In the group assessing names, 57.52% identified as female, with an average age of 37.62 years. The majority ethnicity was White/Caucasian (62.38%), and the prevalent educational attainment was a bachelor's degree (33.91%). In contrast, for the group evaluating categories, females comprised 50.1% of participants, with the largest age group being 25–34 years old (39.1%). This group also showed a higher proportion of White/Caucasian participants (77.7%), and 31.2% had achieved a bachelor's degree.

---

3. I was also wondering what the rationale for using a Prolific sample is here? Would the authors have expected a different result if e.g. the raters had previous experience with hiring decisions?

The following was added to S4:

> Our choice to use a Prolific sample is grounded in literature suggesting that stereotypes are (i) influenced by cultural backgrounds and (ii) pervasively shared within a culture. Consequently,

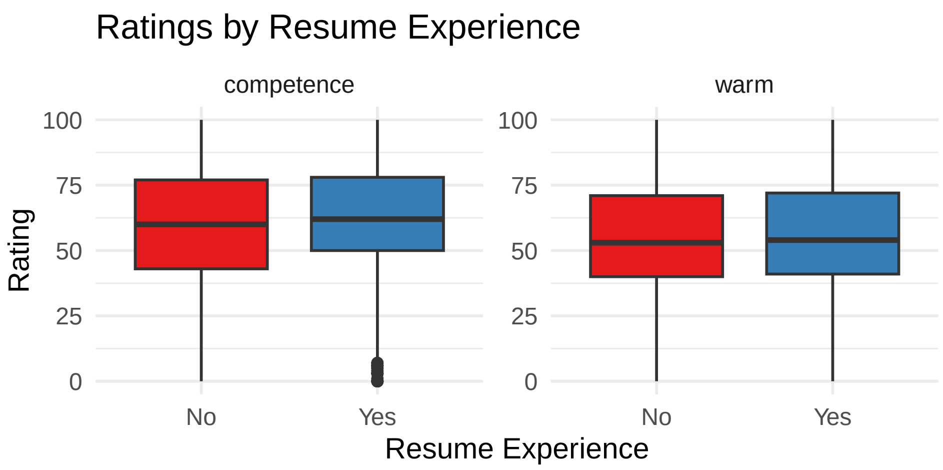


Figure 2: Ratings by resume experience.

individuals with similar cultural backgrounds are likely to hold comparable stereotypes, regardless of their professional background. This assumption holds even when considering participants from varied professions, as our inquiry does not investigate industry-specific perceptions but rather aims to understand societal views on a particular social signal. While one might argue that recruiters, owing to their training, could be less prone to stereotypical assessments in hiring contexts, the question we posed in the online survey transcends specific industries and focuses on broader societal perceptions. The specific wording was: "In your opinion, what does the average American think about this person? Even if you disagree. [signal, e.g. name]" Therefore, we do not anticipate significant variations in responses across samples drawn from different professional backgrounds.

Furthermore, leveraging stereotypes from one sample to predict behaviors in another offers a conservative approach to evaluating the impact of stereotypes on actions. This method likely leads to an underestimation of the effect size compared to directly measuring decision-makers' stereotypes. Our primary concern is with the influence of broad cultural stereotypes on decision-making processes. The extent to which individuals' actions reflect their personal stereotypes, which may not align with societal norms, represents a separate and potentially less consequential issue. This is because societal-level disparities arise when collective decision-making is guided by uniform assumptions based on shared stereotypes.

To address this point more directly, we investigated if prior experience in rating CVs influences warmth and competence judgments among Prolific participants. During our study, Prolific raters (after providing warmth and competence ratings) were asked the following question: "Has it ever been part of your job to evaluate résumés of potential new hires? [yes/no]". 134 raters indicated to not having an experience, 65 raters indicated they had. Given the large sample size of over 7,800 ratings, we were mindful that statistical tests might overemphasize small mean differences. The distributions of ratings between those with and without experience appeared similar upon visual inspection, although Wilcoxon rank-sum tests did indicate significant differences in ratings of warmth (p-value = 1.53e-03) and competence (p-value = 1.317e-08). While statistically significant, these results require future research to assess the practical significance of the differences in ratings.

---

4. On page 5 the authors report "...a marginally significant difference in competence between black and white (-11.52, p = .06)". Considering the massive problems of replication and reproducibility across the behavioral science, I suggest the authors refrain from using such language.

We acknowledge your concern and have removed the mentioned statement.

5. On p. 6, the authors highlight the large prediction interval for the meta-analysis because of the substantial heterogeneity. I think the authors could address this result more in the discussion; for instance, could this result allude to that this literature could benefit from a more standardized approach of testing for labor market discrimination?

We add the following to the discussion:

> The wide prediction interval for the positive correlation in our name analysis suggests that future studies might uncover negative correlations between positive ratings and callback rates. Our stringent selection criteria—restricting studies to those altering names to signify race and gender, conducted in North America, and offering raw data—resulted in a relatively small sample and excluded industry-specific variables. Moreover, our prediction approach adds variability, as it depends on perceptions from a group separate from the actual decision-makers, potentially contributing to the broad prediction interval.

---

6. I was surprised to see that the authors do not test for publication bias in the meta-analysis. If substantial publication bias is present in the current literature, this will naturally influence how much we can trust the findings of the meta-analysis. I think the authors need to conduct analysis of publication bias and correct the estimates for publication-bias.

Because our approach aims to capture the correlation between new (perception ratings) and published data (callback rates), we refrain from using the term "publication bias" since our effect sizes are new and unpublished. However, we report extensive heterogeneity measures analogous to those used in publication bias analyses in SI S2. Specifically, we computed several influence diagnostics (Externally Standardized Residuals, DFFITS Value, Cook's Distance, Covariance Ratio, Leave-One-Out $\tau^2$, Hat Value, Study Weight), which did not nominate any study consistently as an outlier. Furthermore, [1] undertake an analysis to identify any publication bias within their research. They note that while instances of suspected publication bias were addressed by including appropriate caveats within their report, such instances were relatively rare. They specifically point out that the findings related to sexual orientation seemed to be affected by publication bias. Furthermore, they observe that the quantity of correspondence experiments focusing on marital status, wealth, and military service or affiliation was insufficient for drawing robust conclusions. They suggest that areas with scant evidence require further experimental studies to enable researchers to make more definitive statements. In other words, even if there is publication bias, there are too few studies to detect it.

---

7. The exploratory analysis in the last paragraph of section 3 (page 8) only reports the correlation coefficients but not p-values and CI's. I would encourage the authors to report these test statistics to allow the reader a more complete overview of these results.

We appreciate the reviewer's suggestion to include test statistics alongside the correlation coefficients in our exploratory analysis. We have now included robust permutation-based p-values for all reported correlation coefficients in section 3. Our current methodology, however, does not directly yield confidence intervals, a limitation not explicitly addressed within the literature we relied upon for our analytical framework [2, 3]. We acknowledge this gap and believe that the provided p-values will suffice for this exploratory portion of the study.

---

8. Some signals, such as those for wealth and sexuality, yield vastly different ICC's as noted by the authors in the first paragraph of section 4 (page. 8). Why might this be? Could it be a result of certain individual differences in e.g. political orientation etc.? I think it would be valuable if the authors further explore this to identify if there are any systematic influences for these differences. The authors outline this as point of discussion on page 10., but I think they could have elaborated more on this aspect.

Intraclass correlations (ICCs) showed variability across categories, notably in wealth and sexual orientation, prompting a detailed examination (Fig. 3). We analyzed warmth and competence ratings against self-

reported income ranges and sexual orientations of raters. Additionally, we explored socioeconomic status (SES) within studies through indirect signals like preferences for water polo (high SES) versus country or classical music (low SES vs. high SES). Our ANOVA tests found no significant differences in warmth or competence ratings for neither wealth nor sexual orientation. This lack of significance is likely due to the small number of ratings per subgroup, making it difficult to detect meaningful differences. These findings suggest that the variability in ratings is not directly tied to the raters' individual characteristics like income and sexual orientation. This outcome is plausible since raters were instructed to assess how an average American might perceive the subjects. Thus, the ratings reflect the raters' perceptions of societal views rather than personal biases. A comprehensive analysis of these differences and their underlying causes would require an extensive review of related literature and a hypothesis-driven approach, which is beyond this study's scope.

---

9. Lastly, while I agree that the present work can be valuable for the development of responsible AI, I think the authors need to expand on this argument. Right now, this implication comes of as overstated and general.

We expanded upon this point in the discussion as follows:

> Third, our study may contribute to the development of responsible AI by offering computer scientists insights into potential debiasing strategies proven effective in human decision-making, which can be translated into AI models. The literature on responisble AI most frequently measures bias by systematically prompting the model and analyzing the generated or retrieved image output. For example, the text prompt "CEO" will typically be more strongly associated with images of men than women [4, 5]. Most studies rely on sets of examples, e.g., various professions, to detect biases, thereby lacking a validated collection for comprehensively assessing biases. In contrast, an approach grounded in social perception moves beyond sets of examples, providing a broader framework. A few authors in the representation learning literature have seen value in this approach [6, 7, 11, 12]. Specifically, [13] estimate bias in an image dataset by using text attributes of interest from social psychology and creating a set of text prompts. Their results reveal patterns of bias as well as noise in conventional bias measurements.

---

Having mentioned these issues, I wish the authors all the best for their project and I am truly looking forward to reading their revised manuscript, either again for review or published.

Thank you!

---

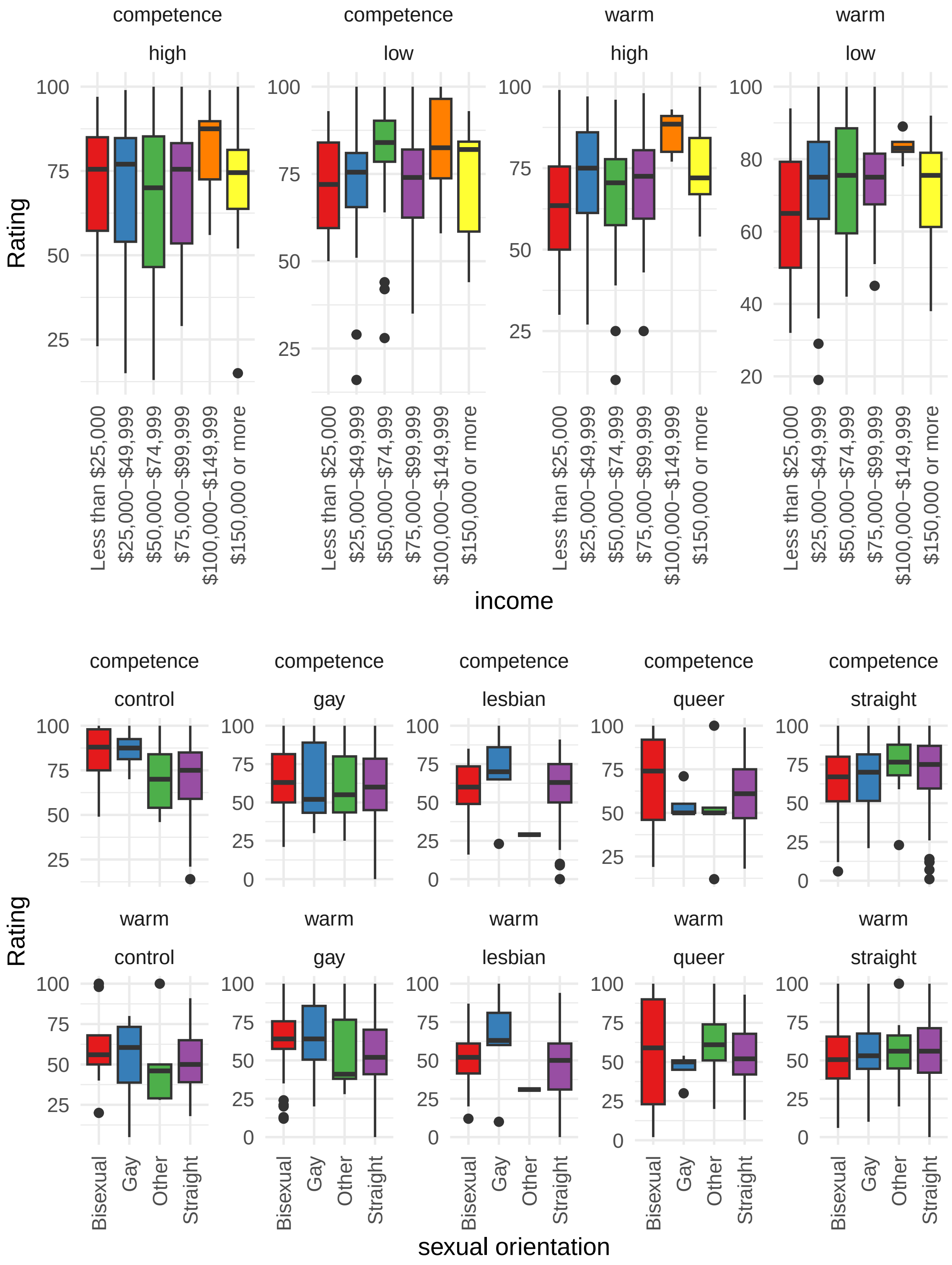


Figure 3: Analysis of Warmth/Competence Ratings by Subgroup. The x-axis represents subgroups defined by participants' self-reported wealth and sexual orientation. Each panel corresponds to different category-signals evaluated. Despite conducting ANOVA tests across these subgroups, no significant differences were observed in ratings based on self-reported metrics. This lack of significance may stem from insufficient sample sizes within each subgroup.

# References

[1] Lippens, Louis, Vermeiren, Siel, and Baert, Stijn. *The State of Hiring Discrimination: A Meta-analysis of (Almost) All Recent Correspondence Experiments.* In *European Economic Review*, 151:104315, 2023. Elsevier.

[2] Yu, Han and Hutson, Alan D. *Inferential Procedures Based on the Weighted Pearson Correlation Coefficient Test Statistic.* In *Journal of Applied Statistics*, 51(3):481–496, February 2024. DOI: 10.1080/02664763.2022.2137477.

[3] DiCiccio, Cyrus J. and Romano, Joseph P. *Robust Permutation Tests for Correlation and Regression Coefficients.* In *Journal of the American Statistical Association*, 112(519):1211–1220, July 2017. DOI: 10.1080/01621459.2016.1202117.

[4] Federico Bianchi, Pratyusha Kalluri, Esin Durmus, Faisal Ladhak, Myra Cheng, Debora Nozza, Tatsunori Hashimoto, Dan Jurafsky, James Zou, and Aylin Caliskan. *Easily Accessible Text-to-Image Generation Amplifies Demographic Stereotypes at Large Scale.* In *2023 ACM Conference on Fairness, Accountability, and Transparency*, pages 1493–1504. 2023. DOI: 10.1145/3593013.3594095.

[5] OpenAi. *Reducing bias and improving safety in DALL · E 2.* July 2022. [Online]. Available: `https://openai.com/blog/reducing-bias-and-improving-safety-in-dall-e-2`. [Accessed: Sept. 21, 2023].

[6] Xubo Cao and Michal Kosinski. *Large Language Models Know How the Personality of Public Figures is Perceived by the General Public.* PsyArXiv preprint, January 2023. DOI: 10.31234/osf.io/89hx6.

[7] Eddie L. Ungless, Amy Rafferty, Hrichika Nag, and Björn Ross. *A Robust Bias Mitigation Procedure Based on the Stereotype Content Model.* arXiv preprint arXiv:2210.14552, October 2022.

[8] Kobayashi, Kenji and Kable, Joseph W and Hsu, Ming and Jenkins, Adrianna C. *Neural representations of others' traits predict social decisions.* Proceedings of the National Academy of Sciences, 119(22):e2116944119, 2022, National Acad Sciences.

[9] Hehman, Eric and Xie, Sally Y and Ofosu, Eugene K and Nespoli, Gabe. *Assessing the point at which averages are stable: A tool illustrated in the context of person perception.* 2018, PsyArXiv.

[10] Jenkins AC, Karashchuk P, Zhu L, Hsu M. Predicting human behavior toward members of different social groups. Proceedings of the National Academy of Sciences. 2018;115(39):9696–9701.

[11] Kathleen C. Fraser, Svetlana Kiritchenko, and Isar Nejadgholi. *A Friendly Face: Do Text-to-Image Systems Rely on Stereotypes when the Input is Under-Specified?* arXiv preprint arXiv:2302.07159, 2023.

[12] Jahna Otterbacher, Jo Bates, and Paul Clough. *Competent Men and Warm Women: Gender Stereotypes and Backlash in Image Search Results.* In *Proceedings of the 2017 CHI Conference on Human Factors in Computing Systems*, pages 6620–6631, Denver Colorado USA, May 2017. ACM. DOI: 10.1145/3025453.3025727.

[13] Hausladen, Carina I. and Knott, Manuel and Camerer, Colin F. and Perona, Pietro. *Measuring social bias in vision-language models using controlled image synthesis.* Working Paper, 2024.
